# Tumor specific liposomes improve detection of pancreatic adenocarcinoma in vivo using optoacoustic tomography

**DOI:** 10.1186/s12951-015-0139-8

**Published:** 2015-12-01

**Authors:** Wenyuan Yin, Charles W. Kimbrough, Jorge G. Gomez-Gutierrez, Christopher T. Burns, Phillip Chuong, William E. Grizzle, Lacey R. McNally

**Affiliations:** University of Louisville, 505 S Hancock, Louisville, KY 40202 USA; University of Alabama Birmingham, ZRB 408, 1720 2nd Avenue South, Birmingham, AL 35294 USA

## Abstract

**Background:**

Pancreatic cancer often goes undiagnosed until late stage disease due in part to suboptimal early detection. Our goal was to develop a Syndecan-1 tagged liposome containing fluorescent dye as an improved contrast agent for detection of pancreatic adenocarcinoma in vivo using multispectral optoacoustic tomography.

**Results:**

The diagnostic capabilities and specificity to pancreatic adenocarcinoma of Syndecan-1 targeted liposomes were evaluated both in vitro and in vivo. Immunocytochemistry showed that liposomes preferentially bound to and released their contents into cells expressing high levels of insulin-like growth factor 1 receptor. We determined that the contents of the liposome were released into the cell as noted by the change in propidium iodide fluorescence from green to red based upon nucleic acid binding. In an orthotopic mouse model, the liposomes preferentially targeted the pancreatic tumor with little off-target binding in the liver and spleen. Peak accumulation of the liposomes in the tumor occurred at 8 h post-injection. Multispectral optoacoustic tomographic imaging was able to provide high-resolution 3D images of the tumor and liposome location. Ex vivo analysis showed that non-targeted liposomes accumulated in the liver, suggesting that specificity of the liposomes for pancreatic adenocarcinoma was due to the presence of the Syndecan-1 ligand.

**Conclusions:**

This study demonstrated that Syndecan-1 liposomes were able to release cargo into IGF1-R expressing tumor cells. The Syndecan-1 liposomes demonstrated tumor specificity in orthotopic pancreatic cancer as observed using multispectral optoacoustic tomography with reduced kidney and liver uptake. By targeting the liposome with Syndecan-1, this nanovehicle has potential as a targeted theranostic nanoparticle for both drug and contrast agent delivery to pancreatic tumors.

**Electronic supplementary material:**

The online version of this article (doi:10.1186/s12951-015-0139-8) contains supplementary material, which is available to authorized users.

## Background

Pancreatic adenocarcinoma has retained a high mortality rate due to inadequate early detection methods and the relative ineffectiveness of current therapy regimens [1–4]. In particular, the underlying biology of pancreatic ductal adenocarcinoma can make these tumors particularly resistant to chemotherapy. The dense desmoplastic stroma and poor vascularity of these tumors inhibits the accumulation of traditional chemotherapies, and prevents therapeutic drug concentrations from being realized within the tumor [5]. Nanoparticle drug carriers are an effective strategy to overcome these extracellular barriers to drug delivery, and can increase the therapeutic load at tumor sites while decreasing systemic toxicity [6–8]. Liposomes in particular have multiple desirable characteristics, such as the capacity for encapsulating large amounts of materials, the ability to protect these materials from degradation, and the capability for intracellular delivery through fusion with the plasma membrane [9].

In addition to being drug carriers, liposomes can also encapsulate contrast agents for diagnostic imaging, combining diagnostic and therapeutic functions into one system. These particles, termed “theranostic nanoparticles”, are a novel approach for detecting, monitoring, and treating cancer [10, 11]. The contrast agents loaded into theranostic nanoparticles allow nanoparticle accumulation and biodistribution to be tracked using standard techniques such as planar fluorescent imaging [12].

However, multi-spectral optoacoustic tomography (MSOT) offers several advantages over traditional imaging modalities. MSOT operates via the photoacoustic effect, wherein a medium excited by pulsed laser light subsequently emits an acoustic wave. Optically active molecules, or chromophores, absorb a photon and enter an excited state, generating heat upon relaxation. This pulse of heat leads to an increase in temperature and local pressure, termed thermoelastic expansion. Propagation of the pressure differential results in the formation of an acoustic wave, which can then be recorded with ultrasound detectors to create high-resolution 3D images [13, 14]. Expansion of endogenous and exogenous chromophores occur at specific excitation wavelengths, allowing for selective imaging of compounds of interest. Due to the low-scattering characteristic of ultrasonic waves, images do not suffer from the light scattering or signal attenuation that limits optical and fluorescent imaging. Additional advantages include an enhanced spatial resolution of up to 150 µm and accurate 3D imaging of deep tissue layers [14–18]. Furthermore, multi-wavelength measurements can be used to quantify spatially varying concentrations of chromophores within biological tissues [13]. MSOT can therefore be a valuable tool in determining the precise location and relative concentration of theranostic nanoparticles in vivo.

Syndecan-1 (Sdc1) has been shown to bind specifically to pancreatic adenocarcinoma, making it a promising targeting ligand for a theranostic nanoparticle [19, 20]. This study examines the feasibility of using Syndecan-1 tagged liposomes as a theranostic nanoparticle for pancreatic adenocarcinoma, using MSOT to evaluate the specificity of these targeted liposomes for pancreatic adenocarcinoma in vivo.

## Methods

### Cell culture

Pancreatic adenocarcinoma cell lines S2VP10 and S2013, metastatic subclones of the SUIT-2 line, were obtained from Dr. Michael A. Hollingsworth at the University of Nebraska. Squamous cell carcinoma cell line SCC-1 was obtained from the University of Michigan. Pancreatic adenocarcinoma cell line MiaPaCa-2 was obtained from ATCC (Manassas, VA, USA). S2VP10, S2013, and SCC-1 cells were cultured in RPMI 1640 and MiaPaCa-2 cells in DMEM medium (Gibco, Grand Island, NY, USA), supplemented with 10 % Fetal Bovine Serum (Atlanta Biologicals, Flowery Branch, GA, USA) and 1 % l-Glutamine (Gibco). The cells were cultured at 37 °C and 5 % CO_2_. S2VP10 cells were infected with a retrovirus containing the firefly luciferase (Luc) gene (Stratagene, La Jolla, CA, USA) and a luciferase-positive single cell clone, S2VP10L, was isolated [21].

### Synthesis of liposomes

#### Materials

Soybean l-α-phosphatidylcholine (95 %) (PC), 1,2-dioleoyl-sn-glycero-3-phosphoethanolamine (DOPE) and 1,2-dioleoyl-sn-glycero-3-phosphoethanolamine-*N*-(hexanoylamine) (CAP) were purchased from Avanti Polar Lipids (Alabaster, AL, USA) (Additional file 1: Figure S1). *N*-Hydroxysulfosuccinimide sodium salt (NHS), *N*-(3-dimethylaminopropyl)-*N*′-ethylcarbodiimide hydrochloride (EDC) and propidium iodide were purchased from Sigma (St. Louis, MO, USA). CF-750 *N*-hydroxysuccinimide ester amine-reactive dye was purchased from Biotium (Hayward, CA, USA). Finally, Syndecan-1 (Sdc1) was purchased from Prospec (Rehovot, Israel).

#### Synthesis of propidium iodide and CF-750 encapsulated liposomes

Naked liposomes were synthesized by thin film hydration method [22]. Soybean PC (0.0067 g), CAP (0.0023 g) and DOPE (0.0020 g) were dissolved in chloroform. Then, the chloroform was evaporated by heating at 70 °C for 10 min in a rotary evaporator to get a thin film of lipids. The thin film was hydrated by adding 1 mL PBS (pH 7.4, 0.9 % NaCl) and propidium iodide or CF-750 (1 mM, 0.1 mL) for 2 h at 70 °C. Then, the liposomes were sonicated for 2 h at 70 °C. Finally, liposomes were extruded four times through a polycarbonate filter (200 nm) at 70 °C.

#### Conjugation of Syndecan-1 to dye-encapsulated liposomes

Syndecan-1 was conjugated to the dye-encapsulated naked liposomes by carbodiimide chemistry. Syndecan-1 (0.74 mM, 20 μl) was mixed with NHS (1 mM, 0.1 mL) and EDC (1 mM, 0.1 mL) and incubated for 6 h at room temperature at pH 7.5. Then, 1 mL of liposomes was added to this solution and mixed for 1 day at room temperature. The excess NHS, EDC, and Sdc1 were removed by dialysis, minimum 2000–3000 Da. The stock solution of Sdc1 conjugated liposomes was diluted further to get the desired concentration.

### Characterization of liposomes

#### DLS measurements

The size distribution of the liposomes was analyzed by dynamic light scattering (DLS) using a Zetasizer Nano-ZS (Malvern Instruments Ltd., Malvern, UK). The detection angle was 173 degrees and the incident beam was a He–Ne ion laser (λ = 633 nm). Correlation functions were analyzed by a histogram method and used to determine the diffusion coefficient (D) of the micelles in the sample. Hydrodynamic radius (Rh) was calculated from D by using Stokes–Einstein’s equation:

$$ R_{h} = \frac{{K_{b} T}}{6\pi \eta D} $$

where kB is Boltzmann’s constant, T is absolute temperature, and η is solvent viscosity [23].

#### Zeta-potential measurements

Electrophoretic mobility (μE) of the liposomes was measured at 25 °C with the Zetasizer Nano-ZS. The zeta-potential of the samples was calculated from μE using following Smoluchowski’s equation:

$$ \mu {\text{E}} = \frac{\zeta \varepsilon }{\eta } $$

where ζ is the zeta-potential, ε the permittivity of solvent, and η the solvent viscosity [23].

#### Transmission electron microscopy

10 μL of 1 mM liposomes were dropped on 200 mesh copper grids (Electron Microscopy Sciences, Hatfield, PA, USA) and dried at room temperature for 5 h. Images were collected using the Phillips CM-10 transmission electron microscope at 80 kV, equipped with a 15 megabyte SIA digital camera.

#### UV–Vis spectroscopy

The absorption spectrum for the CF-750 encapsulated liposomes was analyzed via UV–Visible spectroscopy. Absorption at wavelengths every 10 nm between 650 and 860 nm was measured using the NanoDrop 2000 Spectrophotometer (Thermo Scientific, Waltham, MA, USA) and accompanying software. Near-infrared (NIR) fluorescence signal was confirmed using Li-Cor Odyssey infrared scanner (Li-Cor, Lincoln, NE, USA).

### Western blot analysis

Whole cell lysates from S2VP10L, MiaPaCa-2, S2013, and SCC-1 cells were collected to determine IGF1R expression. The cells were plated at a density of 5x10^5^ cells per well in a 6-well plate 24 h prior to protein harvest. Cells were then lysed in a solution containing 1 % NP-40, 1 % protease inhibitor, and 1 % phosphatase inhibitor (Thermo) in deionized water. The lysates were centrifuged at 13,000×*g* for 10 min. Total protein concentration was determined via Bradford assay (Bio-Rad, Hercules, CA, USA).

Approximately 50 µg of total protein was dissolved in deionized water, loading buffer, and reducing agent (Life Technologies, Carlsbad, CA, USA). Proteins were separated using NuPage 4–12 % Bis–Tris gel and transferred onto a nitrocellulose membrane by iBlot (Life Technologies). The membrane was blocked in blocking buffer (Li-Cor) for 30 min and then incubated overnight at 4 °C with IGF1R antibody (Abcam, Cambridge, England) at a concentration of 1 µg/mL and β-Actin antibody (Thermo) at a concentration of 1:3000. The membrane was then washed 3× with TBS (20 mM Tris–HCl, 150 mM NaCl in diH_2_O) for 10 min each, incubated with donkey anti-mouse IRDye 680RD and donkey anti-rabbit IRDye 800CW secondary antibodies (Li-Cor) for 1 h, washed 3× with TBS for 10 min each, and scanned using Li-Cor Odyssey infrared scanner. Dosimetry was performed using Li-Cor software.

### Immunocytochemistry

S2VP10L and SCC-1 cells were each seeded into 3 wells of a chamber slide at a density of 3 × 10^5^ cells/well. Cells were grown overnight in RPMI with 10 % FBS and 1 % l-Glutamine at 37 °C with 5 % CO_2_. The cells were then serum-starved for 3 h by switching to RPMI with 1 % BSA (Fisher). Immunocytochemistry buffers were prepared as follows: PBS^++^ (0.5 mM CaCl_2_ and MgCl_2_ in PBS), PBS^++++^ (97.9 mL PBS++, 2 mL 10 % BSA+ 90.08 mg dextrose), and citrate buffer (1 mL 20× SCC buffer, 19 mL ddH_2_O). Following starvation, cells of each line were treated with 30 μL PBS (control), 30 μL of 1 mM naked liposomes, or 30 μL of 1 mM Sdc1 liposomes for 3 h. Then, the wells were washed 2× with cold PBS^++++^, 3× with ice cold citrate buffer, and again 2× with cold PBS^++++^. Cells were fixed with 4 % paraformaldehyde (Electron Microscopy Sciences) for 5 min at room temperature and 15 min at 4 °C. Next, the cells were washed 6× with PBS^++^ for 10 min each. The slides were mounted in ProLong with DAPI (Invitrogen, Waltham, MA, USA) and set overnight. Images were taken at 400× magnification with a Zeiss Image Photomicroscope 2 (Carl Zeiss, Oberkochen, Germany) using DAPI, FITC, and Texas Red filters. The exposure times used were 20 ms DAPI, 200 ms FITC, and 200 ms Texas Red.

### Orthotopic pancreatic cancer xenografts

Strict adherence to the University of Louisville Institutional Animal Care and Use Committee (IACUC)-approved protocol was maintained throughout the study. Five-week old female CB-17 SCID mice (Harlan Laboratories, Indianapolis, IN, USA) were acclimated for 1 week prior to the study. Mice were anesthetized for all surgical procedures with 1.6 % isoflurane and all procedures were performed in a sterile hood. Orthotopic cell implantation was performed as previously described [15, 21]. A 1-cm incision was made in the left upper abdominal quadrant and the spleen was located and used to indirectly position the tail of the pancreas, avoiding direct manipulation of the pancreas. The suspension of S2VP10L (Luc positive) cells in PBS was stored on ice and drawn up with a 28-gauge needle. Five mice were each injected with 1.5 × 10^5^ S2VP10L (Luc positive) cells (30 µL) into the tail of the pancreas. Sterile cotton-tipped applicators were used to cover the injection site for 30 s to prevent peritoneal leakage. The organs were repositioned in the abdomen and the incision was closed using a single-layer closure with 5-0 nylon sutures. The mice recovered on a heated pad while receiving clear liquid acetaminophen for 24 h with food and water ad libitum. Confirmation of orthotopic implantation was performed using bioluminescence optical imaging on the Advanced Molecular Imager AMI-1000X (Spectral Imaging Instruments, Tucson, AZ, USA). Mice with detectable leakage from the pancreas were removed from the study. Mice received intraperitoneal (IP) injection of 2.5 mg luciferin 10 min prior to weekly imaging to monitor orthotopic tumor and metastatic growth. Region of interest (ROI) analysis was used to measure light emitted for orthotopic sites using the AMI Image Viewer software. Sutures were removed 7 days following implantation. At 10 days post-implantation, mice were IV injected with 200 μL of 100 nM untargeted CF-750 or Sdc1-tagged CF-750 liposomes.

### In vivo imaging and reconstruction

Multispectral optoacoustic tomographic (MSOT) imaging was performed as described by Kimbrough et al. [20]. Mice were anesthetized with 1.6 % isoflurane and prepped for imaging with a combination of Nair cream with aloe (Church and Dwight Co., Princeton, NJ, USA) and shaving. The mice were imaged using MSOT system InVision TF 256 (iThera Medical, Munich, Germany). Serial slice images were taken in 0.2 mm steps between the diaphragm to the bottom of the kidneys (40–55 mm) at wavelengths of 680, 710, 730, 740, 760, 770, 780, 800, 850, and 900 nm using 25 averages per wavelength with acquisition time of 10 µs per frame [15, 16] in order to minimize the influence of animal movement. Images were taken at 4, 8, 16, and 24 h post injection to track liposome uptake and accumulation. Excitation of CF-750 liposomes was conducted using a tunable parametric oscillator pumped by an Nd:YAG laser. The pancreas tumor was identified by a live-feed screen preview multispectral signal. Video-rate acquisition produced an imaging clip compiled from single-slice acquisition in less than 1 ms, resulting in an image data rate of 10 frames per second. Image reconstruction was conducted using backprojection at a resolution of 75 µm. Multispectral processing was conducted using linear regression (ViewMSOT 3.5). A 3.5 mm diameter ellipse was used for region-of-interest analysis of liposome signal for tumor, liver, and kidney as in [15].

### Ex vivo imaging of organs

Mice were euthanized 24 h after liposomal injections via carbon dioxide inhalation. The pancreas, liver, and spleen were harvested and imaged ex vivo to compare accumulation of naked and Sdc1 tagged liposomes from each of the five mice imaged using MSOT. The organs were imaged using near-infrared (NIR) fluorescence with the AMI-1000X at 675 nm excitation and 760 nm emission.

## Results and discussion

### Western blot

Western blot analysis was performed to evaluate the expression of IGF1-R on the three different pancreatic adenocarcinoma cell lines (S2VP10L, MiaPaCa-2, and S2013). The head and neck squamous cell carcinoma cell line SCC-1 served as a negative control. IGF1R plays an important role in cell proliferation, tumor metastasis, and anti-apoptotic pathways and thus is highly expressed in aggressive cancers. High IGF1R expression correlates to resistance to chemotherapy and radiation-induced apoptosis and is a marker for poor prognosis in pancreatic, breast, lung, and prostate cancers [24–29].

**Fig. 1:**
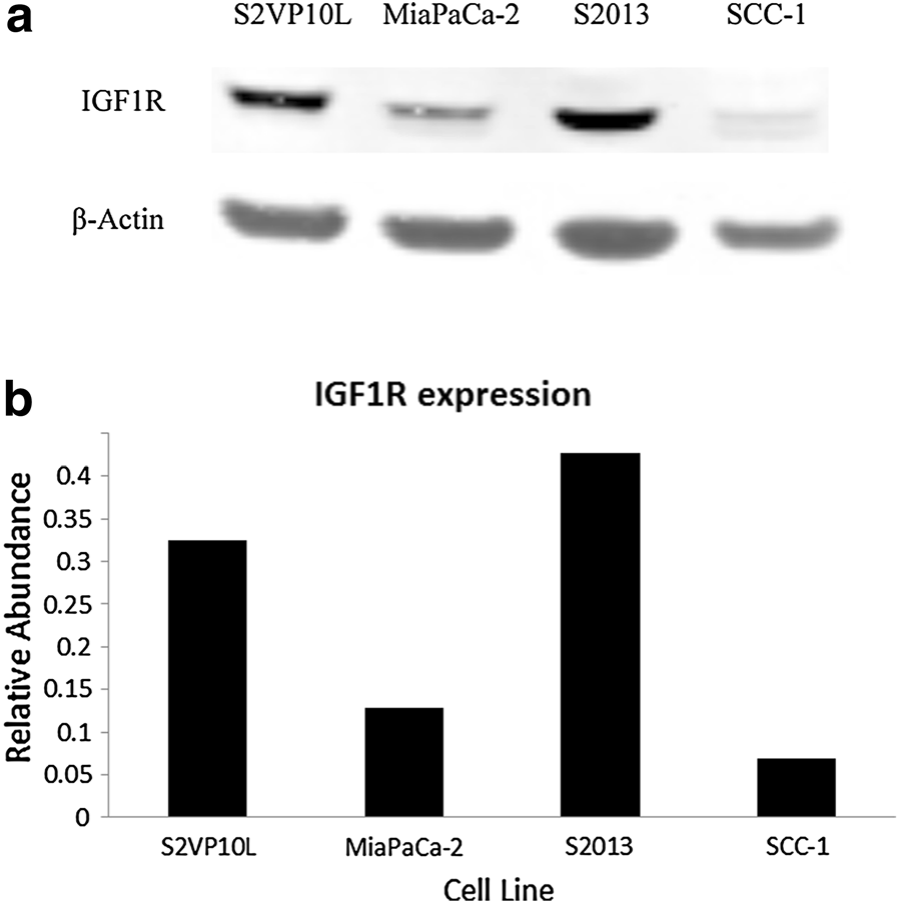
Western blot analysis of pancreatic cell lines S2VP10L, MiaPaCa-2, S2013, and head and neck squamous cell carcinoma cell line SCC-1. **a** Western blot bands of β-Actin (42 kDa) and IGF1R (90 kDa). **b** Western blot band intensity dosimetry. The signal intensity of the IGF1R band was divided by the signal intensity of the β-Actin band to calculate relative abundance. Relative abundance of IGF1R in negative control SCC-1 cells was approximately 0.06. Pancreatic cell lines S2VP10L and S2013 displayed relative abundance of IGF1R of 0.32 and 0.42, respectively, a five- and seven-fold increase compared to the negative control

As expected, SCC-1 cells expressed much lower levels of IGF1R than pancreatic cancer lines (Fig. 1a). Less aggressive MiaPaCa-2 cells also had relatively low IGF1R expression. Highly aggressive and metastatic S2VP10L and S2013 cells had five- and seven-fold increase of IGF1R expression, respectively compared to SCC-1 cells (Fig. 1b). S2VP10L cells were chosen as the pancreatic cell line to be used for the remainder of the experiments due to their predictable behavior in vivo and relatively high levels of IGF1R expression.

Syndecan-1 (Sdc1) is a transmembrane heparin sulfate proteoglycan that plays a role in cell proliferation and migration. The ectodomain of Sdc1 interacts with IGF1R to activate α_5_β_3_ integrin, a mechanism not found in non-malignant epithelial cells [24, 30, 31]. High IGF1R expression in aggressive cancers coupled with the Sdc1–IGF1R–α_5_β_3_ integrin interaction makes targeting IGF1R via Sdc1 an attractive option for a theranostic nanoparticle for pancreatic adenocarcinoma.

### Characterization of liposomes

**Fig. 2:**
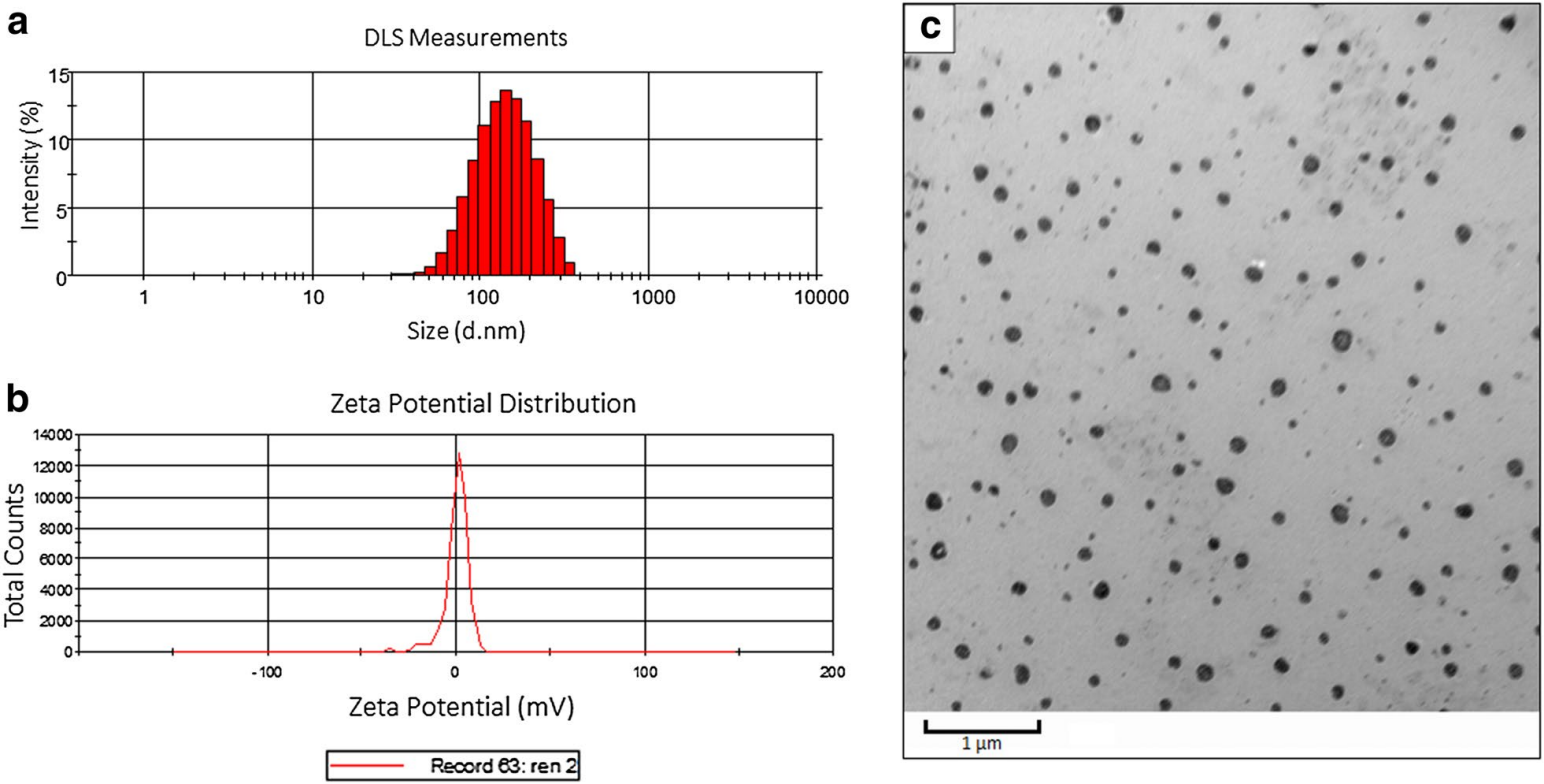
**a** DLS measurements to determine the size of the liposomes. The liposomes are approximately 129 nm in diameter with a polydispersion index of 0.05. **b** Zeta-potential measurements of the liposomes. Z = 0.3 mV. **c** Transmission electron microscopy of the liposomes. The average size of the liposomes as determined by TEM is approximately 117 nm

Liposomes were synthesized to encapsulate CF-750 dye or propidium iodide and tagged with Syndecan-1. Transmission electron microscopy (TEM) of the liposomes shows the average size to be 117 nm (Fig. 2c). The size of liposomes observed by TEM is smaller than DLS (129 nm, Fig. 2a), as TEM yields a number-averaged size whereas DLS does a Z-averaged size that takes into account hydration layers. Based on the low polydispersion index, the liposomes are uniform in size (Fig. 2a). The liposomes also have a slight positive charge of 0.3 mV (Fig. 2b).

The leaky vasculature and inadequate lymphatics around solid tumors encourages accumulation of nanoparticles of a particular size (~100 nm), a phenomenon termed the enhanced permeability and retention effect (EPR) [32]. It is estimated that nanoparticles 60–150 nm are small enough to extravasate from the blood into the tumor interstitial space through these pores within the vasculature [33]. Slightly larger particles such as albumin-bound chemotherapies with diameters around 130 nm also display EPR and are suitable for IV injection [34]. Liposomal nanocarriers such as Sdc1-tagged liposomes can use passive targeting via EPR to deliver therapeutic agents specifically to tumor cells while bypassing normal, healthy tissue. Treatment with liposome-encapsulated therapeutics have been shown bioavailability of therapeutic agents at the tumor site, reduces off-target toxicity, and increases circulation time [35, 36]. Additionally, liposomes are permeable in environments with high collagen content, a characteristic of the extracellular matrix of pancreatic tumors, making them particularly attractive nanoparticles for pancreatic adenocarcinoma [37, 38].

In this study, Syndecan-1 was conjugated to the liposomes to act as a targeting ligand, a molecule that interacts with extracellular or trans-membrane molecules on cancer cells [19]. This allows Sdc1-tagged liposomes to take advantage of EPR and active targeting of IGF1R to enhance specificity for the tumor. Receptor-ligand targeting has been shown to improve target cell recognition within the tumor, leading to increased accumulation within cancer cells and increased cellular uptake when compared to non-targeted particles [39].

#### UV–Vis spectroscopy

The absorption spectrum for CF-750 encapsulated Sdc1 liposomes was analyzed using UV–Vis spectroscopy. The peak absorption of the liposomes was 750 nm, the same as the original dye (Additional file 2: Figure S2). Thus, encapsulating the dye within liposomes does not change the optical behavior of the dye.

### Immunocytochemistry

**Fig. 3:**
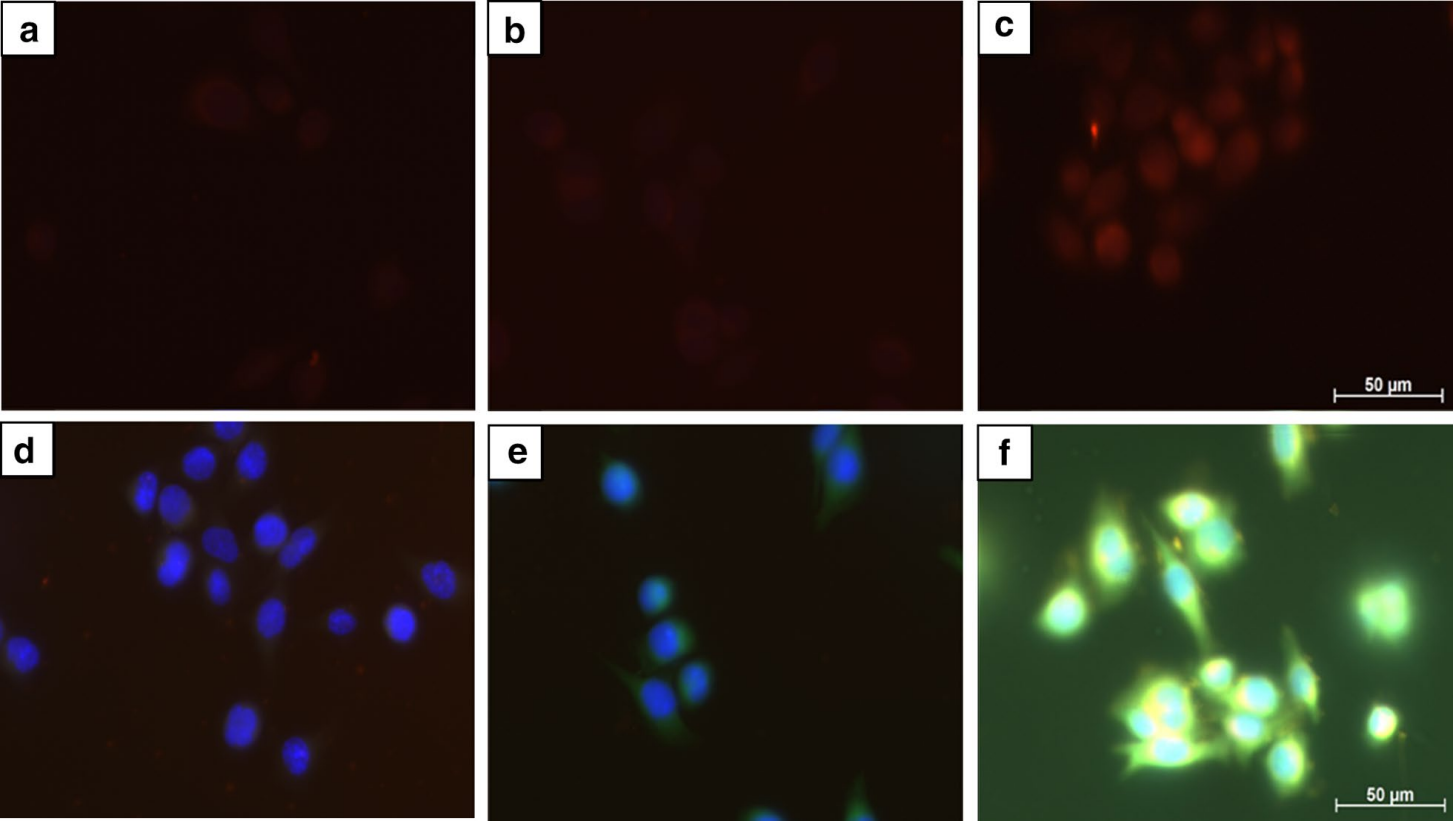
S2VP10L cells following 3 h treatment at ×400 magnification. All images were taken with the same exposure times. *Top row* **a**–**c** was taken with the Texas Red filter. *Bottom row* **d**–**f** was taken with Texas Red, DAPI, and FITC filters. **a** Untreated control cells show minimal *red* autofluorescence. **b** Cells were treated with non-targeted liposomes display faint *red* signal, corresponding to minimal dye uptake. **c** Cells were treated with Sdc1 liposomes and a much stronger *red* fluorescence, indicating uptake of PI and binding of PI to DNA is observed. **d** Control cells with DAPI. **e** Cells treated with non-targeted liposomes. *Green* signal is due to unbound PI, showing dye accumulation outside of the cell. **f** Cells treated with Sdc1 liposomes. *Yellow* signal occurs due to co-localization of *red* and *green* fluorescence, indicating that dye was located both on the cell surface and in the cytoplasm. *White* signal is colocalization of red, green, and DAPI signal

**Fig. 4:**
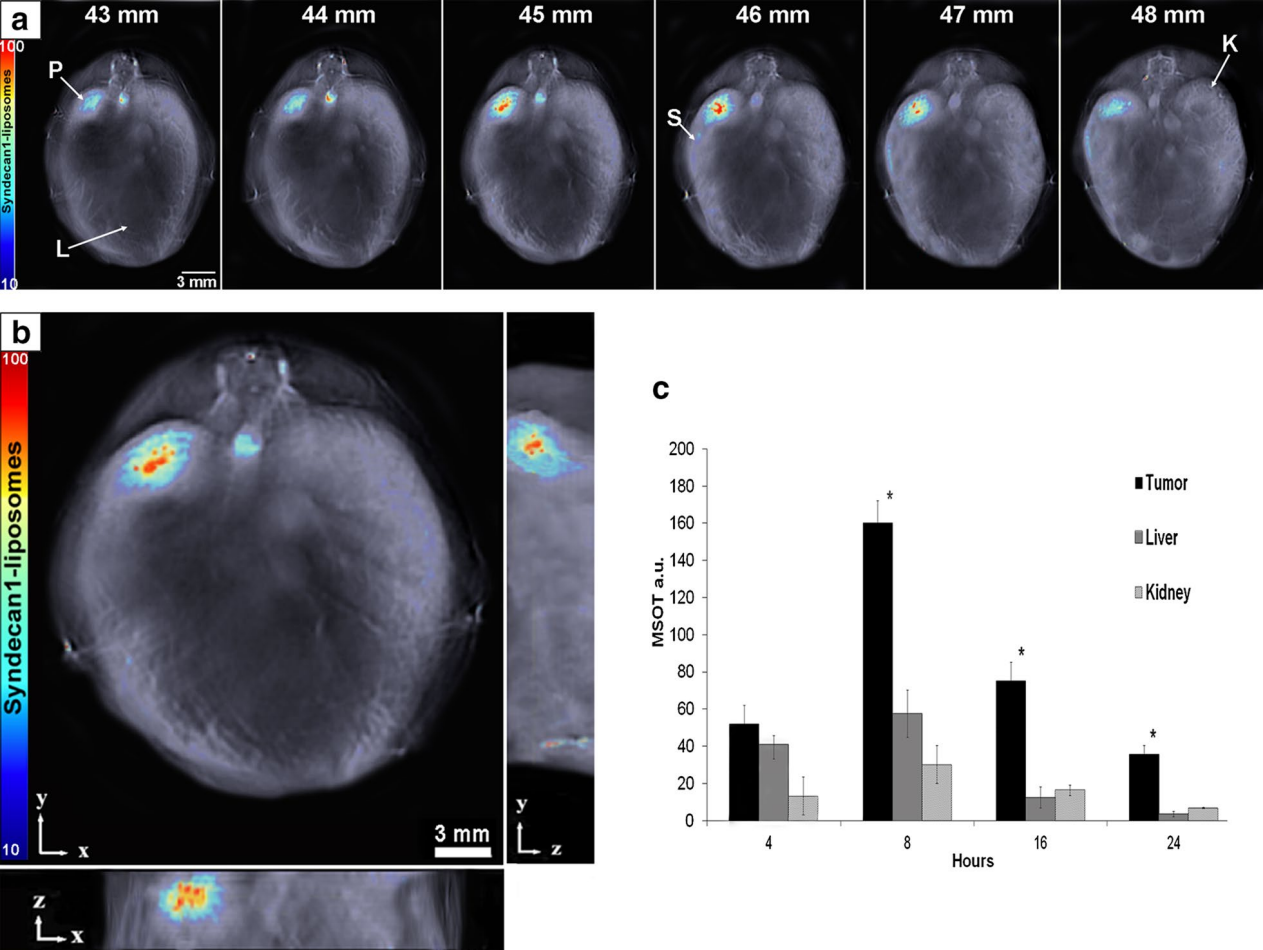
**a** Serial slice images of liposomal accumulation taken from 43 to 48 mm (abdomen). The highest signal intensity of the liposomes was at 45–46 mm. Organs are identified: *P* pancreas tumor, *L* liver, *S* spleen, *K* kidney. **b** Orthogonal views of the pancreatic tumor and liposomal accumulation through different anatomical planes. **c** ROI analysis on liposome signal in various locations over time measured in MSOT a.u. *Bar* height represents the median value and *error bars* represent the standard deviation throughout the organ. Peak liposomal accumulation occurred at 8 h post-injection. Significantly more liposomes accumulated in the tumor versus off-target organs (p < 0.05). Representative locations of organs can be viewed in Additional file 3: Figure S3

Immunocytochemistry slides of S2VP10L and SCC-1 cells were treated with non-targeted and Sdc1 liposomes containing propidium iodide and imaged using Texas Red, DAPI, and FITC filters. Fluorescence seen using the Texas Red filter indicates propidium iodide (PI) uptake by the cell. In IGF1R-positive S2VP10L cells, we found greatly increased uptake of PI when treated with Sdc1-tagged liposomes (Fig. 3c) compared to naked liposomes (Fig. 3b). Treatment with non-targeted liposomes does not result in the intracellular localization of dye, as the green fluorescence indicates extracellular accumulation (Fig. 3e) rather than intracellular uptake. The bright yellow areas in Fig. 4f indicate locations where red and green light are both present, suggesting PI on both the cell surface as well as within the cytoplasm, where it is likely bound to mitochondrial DNA or free-floating RNA. White areas indicate red, green, and blue co-localization demonstrating the PI binding to nuclear DNA. Untreated S2VP10L cells show minimal red autofluorescence (Fig. 3a) and no green autofluorescence (Fig. 3d).

Propidium iodide, when bound to nucleic acids, displays maximum excitation and emission values at 538 and 617 nm, respectively and fluoresces red using the Texas Red filter. However, PI in its free form exhibits different optical characteristics: maximum excitation at 488 nm and emission at 590 nm [40], fluorescing green using the FITC filter. This allows for distinction between excess dye bound on the cell surface versus internalized dye bound to nuclear or cytoplasmic nucleic acids.

S2VP10L cells treated with Sdc1 liposomes show dramatically increased PI uptake compared to cells treated with non-targeted liposomes. The Sdc1–IGF1R interaction may facilitate the liposomes to release their contents into the cell. Since this mechanism is specific to IGF1R-positive cells, chemotherapeutic agents may be loaded into Sdc1 liposomes and delivered only to tumor cells. Using a nanocarrier of this sort can reduce the incidence and intensity of side effects, which results in improved patient compliance and prognosis [6–8, 10]. Furthermore, the presence of a contrast agent allows for tracking of liposomal movement and accumulation and can be used to diagnose pancreatic cancer. Thus, Sdc1-tagged liposomes have the potential to fulfill both diagnostic and therapeutic functions.

### In vivo imaging and reconstruction

Syndecan-1-tagged liposomes were injected 10 days post-implantation of the tumor. Liposome accumulation in vivo was determined using MSOT imaging at 4, 8, 16, 24 h post injection. Representative location of organs as seen on the MSOT can be viewed in Supplemental Fig. 3. Liposome accumulation in the tumor peaked at 8 h post-injection and declined within 24 h (Fig. 4c). The highest region of photoacoustic signal was between 45 and 46 mm in the mouse, and corresponded with the location of the pancreatic tumors (Fig. 4a). Low signal intensity was observed in the spleen and liver (Fig. 4b), indicating significantly more liposomes accumulated in the tumor versus in the liver and kidney (p < 0.05). Furthermore, orthogonal views of the tumor demonstrate probe accumulation along three spatial dimensions, and suggest liposome penetration and accumulation within the interior of the pancreatic tumor (Fig. 4b). Error bars represent standard deviation throughout the organ (Fig. 4c).

The ability to utilize targeting ligands or monoclonal antibodies to improve tumor specific uptake of nanoparticles for both imaging and treatment of cancer is one of the most important aspects to successfully translate nanotechnology to the clinic. This is especially important as non-targeted liposomal formulations of chemotherapeutics, i.e. doxil, have failed to demonstrate significantly increased tumor localization in the clinic [41]. The use of Syndecan-1 ligand to target the liposomes to the pancreatic tumor along with low off-target binding demonstrates the feasibility of this approach. The ability of these particles to contain contrast agents can allow for alternative methods of imaging pancreatic cancer using current imaging technology. Previous studies have shown that liposome-encapsulated contrast agents enhance imaging of tumors and inflammatory lesions using CT and SPECT by increasing circulation time [42, 43]. Modifying the lipid composition of Sdc1-tagged liposomes in this way could enhance the use of MRI for pancreatic adenocarcinoma by allowing for the imaging of smaller tumors. Alternatively, Sdc1-tagged or similar liposomes specific to pancreatic cancer can be loaded with a contrast dye to better image suspected pancreatic lesions using CT, improving an already-established method of imaging pancreatic cancer.

Photoacoustic imaging is capable of producing high resolution molecular images in vivo based on the absorption of contrast agents. MSOT technology, while new, is being established in clinical settings with the development of a handheld MSOT system [44]. In humans, MSOT has been able to provide high-resolution and high-contrast imaging of both endogenous (such as oxy- and deoxy-hemoglobin, and melanin in the monitoring of melanoma) and exogenous (NIR dyes) chromophores in the context of cancer [45–47]. Currently, photoacoustic imaging has been demonstrated at depths of up to 5 cm while volumetric real-time photoacoustic imaging image has been demonstrated depths of up to 1 cm [46].

The use of photoacoustic imaging of pancreatic cancer is currently limited by inability to deliver the laser pulse to deep organs. However, photoacoustic endoscopy, currently in the preclinical stage, is emerging as a new modality for imaging the GI tract [48]. Endoscopic ultrasound is currently used for diagnosis of pancreatic cancer via ultrasound-guided needle biopsies of suspected tumors [4]. With further technological advancement, photoacoustic endoscopy can deliver light through the lining of the stomach to the pancreas. If used to image pancreatic adenocarcinoma-specific chromophores, such as dyes encapsulated within targeted liposomes, photoacoustic endoscopy has the potential to become a non-invasive method of diagnosing pancreatic cancer.

### Ex vivo imaging of organs

**Fig. 5:**
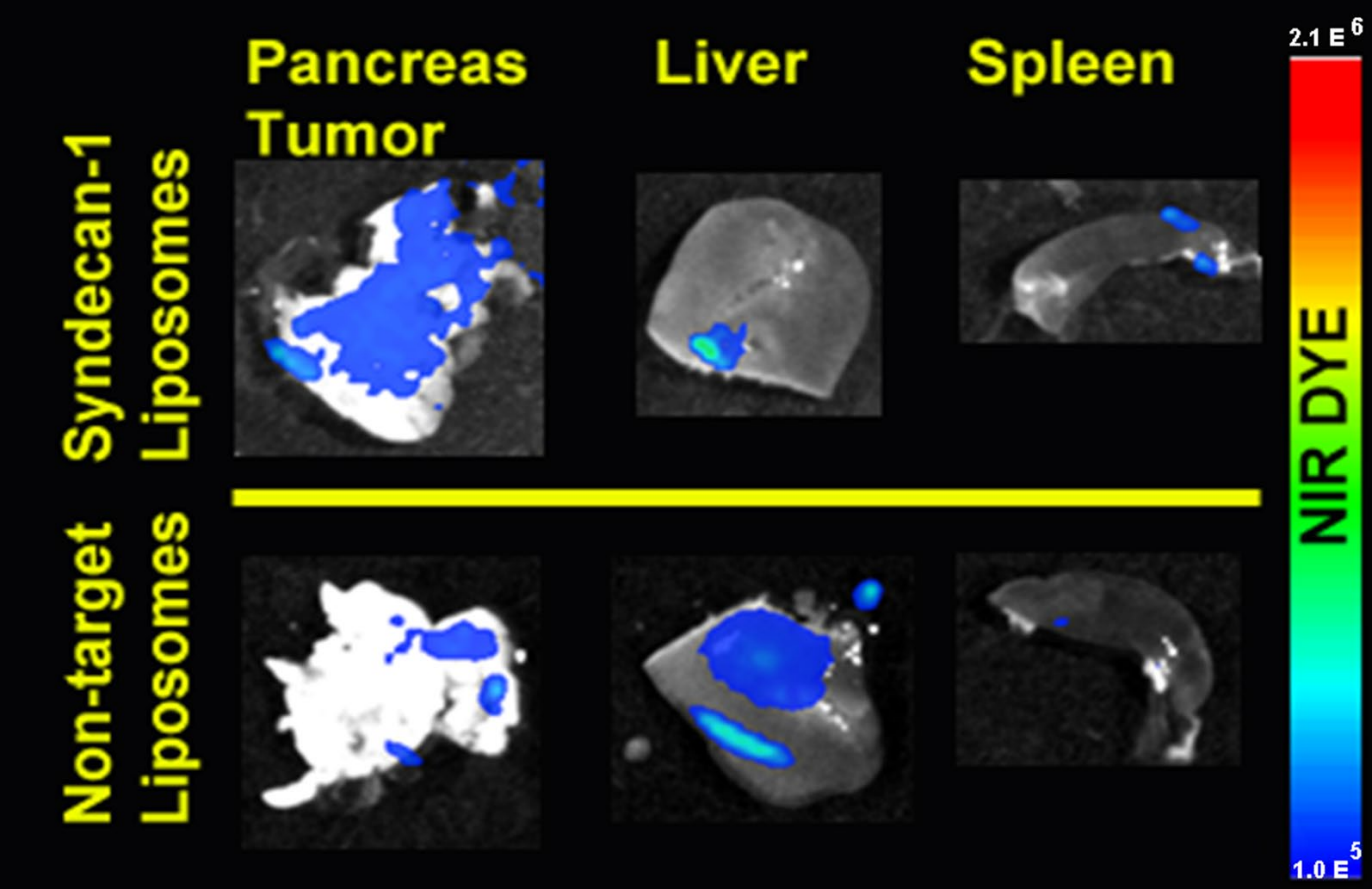
Ex vivo fluorescent imaging of the pancreas, liver, and spleen 24 h post-injection. Sdc1 liposomes accumulated in the tumor with very little off-target binding in the liver and spleen. Non-targeted liposomes tend to accumulate in the liver with low signal in the tumor

Ex vivo fluorescent imaging confirms accumulation of Sdc1-liposomes within organs (Fig. 5). Mice were euthanized 24 h post-liposomal injection. The pancreas, liver, and spleen were imaged using NIR fluorescent imaging with excitation 675 nm and emission 760 nm. Sdc1-tagged liposomes bound preferentially to the pancreas tumor with little off-target binding in the liver and spleen. While some naked liposomes reached the pancreatic tumor, this was likely due to passive targeting from EPR and the leakiness of the blood vessels around the tumor [32]. Non-targeted liposomes accumulated primarily within the liver.

The dramatic difference in liposome localization and specificity was due to the presence of the targeting ligand, Syndecan-1. Sdc1 provided a mechanism for the liposomes to actively target pancreatic cancer by binding to IGF1R, overexpressed on S2VP10L cells (Fig. 1). Active targeting enhanced the specificity of the liposomes for pancreatic cancer when compared to untargeted liposomes, which is in congruence with previous studies [39].

## Conclusion

Syndecan-1 tagged liposomes actively target pancreatic adenocarcinoma with minimal off-target binding in vivo. Using liposomes that contained contrast agents allowed for non-invasive tracking via photoacoustic imaging, and MSOT was able to provide high-resolution molecular images of our Syndecan-1 liposomes in an orthotopic pancreatic xenograft mouse model. *Ex vivo* analysis of non-targeted and Syndecan-1 liposomes show that unlike naked liposomes, Syndecan-1 liposomes do not primarily accumulate in off-target organs such as the liver. In this study, the liposomes only contained a fluorescent dye for diagnostic imaging; in the future, these liposomes may serve as a nanocarrier for chemotherapeutic agents. Our in vitro results demonstrate improved intracellular delivery of liposomal content into tumor cells using targeted liposomes. Future studies should explore the ability of Syndecan-1 liposomes to preferentially release drugs at the tumor site and compare their effectiveness to current treatments for pancreatic cancer. Ultimately these studies may lead to the development of a theranostic nanoparticle for clinical use.

## Additional files

10.1186/s12951-015-0139-8  Structures of the lipids used for control and Sdc1-tagged liposome synthesis.
10.1186/s12951-015-0139-8 Absorption spectrum for CF-750 encapsulated Sdc1 liposomes. The liposomes demonstrated fluorescence activity with peak absorbance at 750 nm. Encapsulating the CF-750 dye within the Sdc1 liposomes did not change the optical activity of the dye.
10.1186/s12951-015-0139-8  Representative locations of organs on MSOT at both 46 and 49 mm. Organs are noted PT = Pancreas tumor, S = Spleen, L = Liver, BV = Blood vessel, K = Kidney.
