# Supplementary figures and images for: Tumor specific liposomes improve detection of pancreatic adenocarcinoma in vivo using optoacoustic tomography
Source: J Nanobiotechnology. 2015 Dec 1;13:90. doi: 10.1186/s12951-015-0139-8 (PMC4665906; doi:10.1186/s12951-015-0139-8)

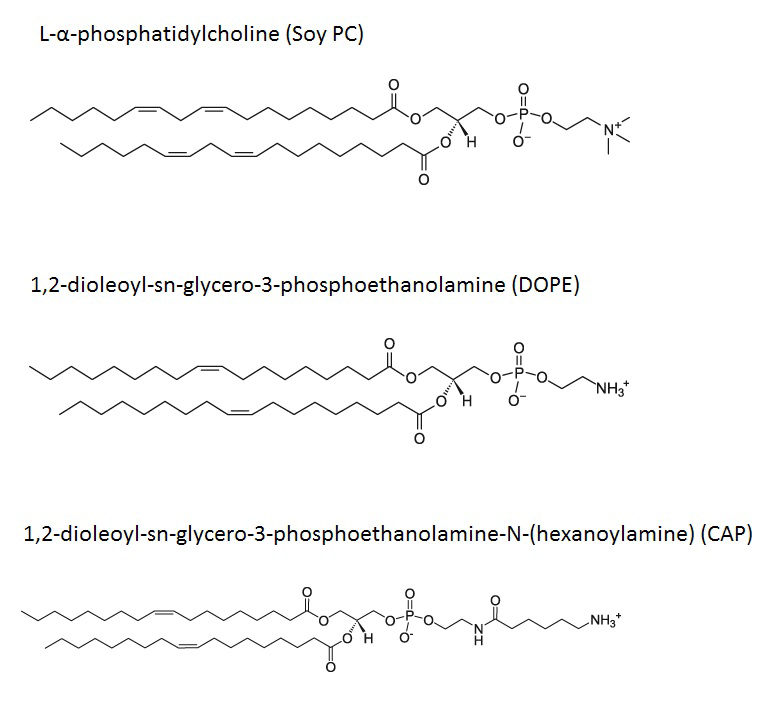

Supplement: Supplementary file 1 — 10.1186/s12951-015-0139-8 Structures of the lipids used for control and Sdc1-tagged liposome synthesis. [file 12951_2015_139_MOESM1_ESM.tif]

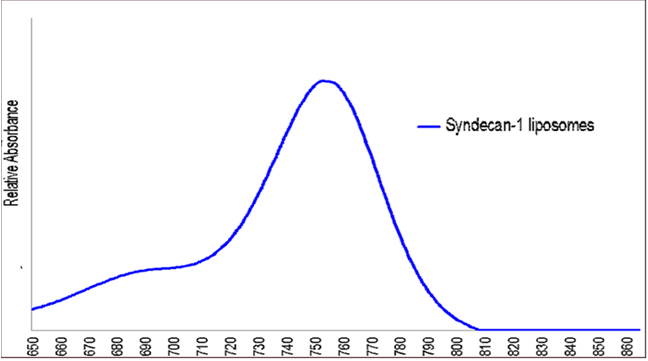

Supplement: Supplementary file 2 — 10.1186/s12951-015-0139-8 Absorption spectrum for CF-750 encapsulated Sdc1 liposomes. The liposomes demonstrated fluorescence activity with peak absorbance at 750 nm. Encapsulating the CF-750 dye within the Sdc1 liposomes did not change the optical activity of the dye. [file 12951_2015_139_MOESM2_ESM.tif]

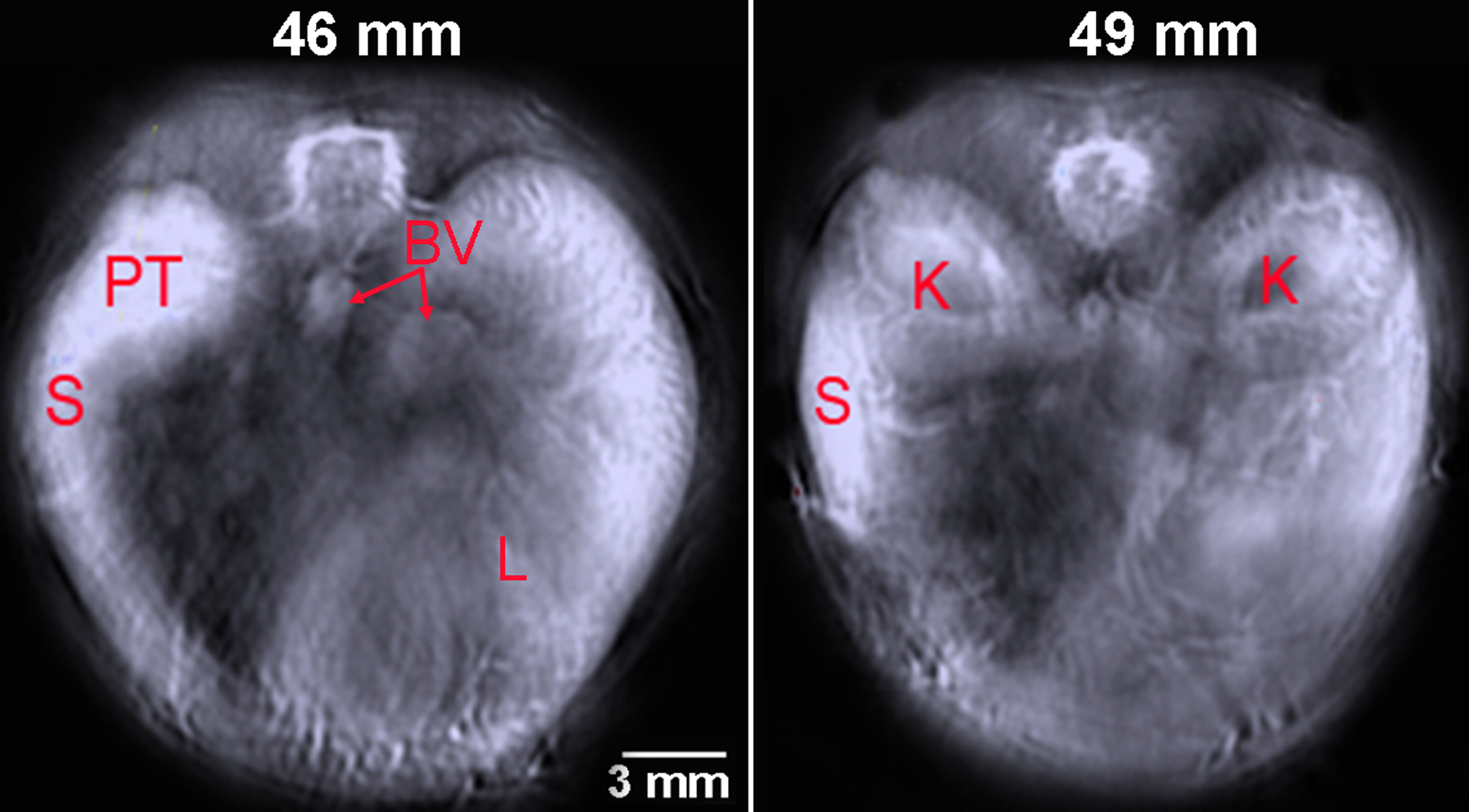

Supplement: Supplementary file 3 — 10.1186/s12951-015-0139-8 Representative locations of organs on MSOT at both 46 and 49 mm. Organs are noted PT = Pancreas tumor, S = Spleen, L = Liver, BV = Blood vessel, K = Kidney. [file 12951_2015_139_MOESM3_ESM.tif]
